# 3-Anilino-*N*-*p*-tolyl­benzamide

**DOI:** 10.1107/S1600536809049939

**Published:** 2009-12-12

**Authors:** Xing-Xing Yang, Guan-Feng Liu, Da-Bin Qin

**Affiliations:** a School of Chemistry and Chemical Engineering, China West Normal University, Nanchong 637002, People’s Republic of China

## Abstract

The title compound, C_20_H_18_N_2_O, which crystallizes with two independent mol­ecules (*A* and *B*) in the asymmetric unit, is composed of three aromatic rings (I, II and III). The conformation of the two independent mol­ecules is slightly different. The dihedral angles between the central aromatic ring II and rings I and III are 47.13 (9) and 89.36 (9)°, respectively, for mol­ecule *A*, and 29.60 (9) and 70.72 (9)°, respectively, for mol­ecule *B*. Rings I and III are inclined to one another by 86.57 (9)° in mol­ecule *A*, and 64.59 (10)° in mol­ecule *B*. The mol­ecular structures are stabilized by intra­molecular N—H⋯O hydrogen bonds. In the crystal structure, mol­ecules are linked through inter­molecular N—H⋯O hydrogen bonds, forming chains propagating in the [010] direction. In addition, a number of C—H⋯π inter­actions are observed.

## Related literature

For the synthesis, see: Martín *et al.* (2006 ▶); Charton *et al.* (2006 ▶). For related structures, see: Du *et al.* (2009 ▶); Qi *et al.* (2002 ▶)..
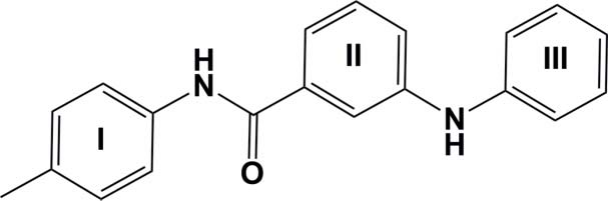


## Experimental

### 

#### Crystal data

C_20_H_18_N_2_O
*M* *_r_* = 302.36
Orthorhombic, 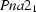

*a* = 26.537 (3) Å
*b* = 17.7337 (19) Å
*c* = 6.8457 (7) Å
*V* = 3221.6 (6) Å^3^
*Z* = 8
Mo *K*α radiation
μ = 0.08 mm^−1^
*T* = 93 K
0.50 × 0.40 × 0.33 mm

#### Data collection

Rigaku SPIDER diffractometer
Absorption correction: none
21180 measured reflections
3989 independent reflections
3913 reflections with *I* > 2σ(*I*)
*R*_int_ = 0.028

#### Refinement

*R*[*F* ^2^ > 2σ(*F* ^2^)] = 0.034
*wR*(*F* ^2^) = 0.074
*S* = 1.01
3989 reflections
433 parameters
1 restraint
H atoms treated by a mixture of independent and constrained refinement
Δρ_max_ = 0.20 e Å^−3^
Δρ_min_ = −0.14 e Å^−3^

### 

Data collection: *RAPID-AUTO* (Rigaku/MSC, 2004 ▶); cell refinement: *RAPID-AUTO*; data reduction: *RAPID-AUTO*; program(s) used to solve structure: *SHELXS97* (Sheldrick, 2008 ▶); program(s) used to refine structure: *SHELXL97* (Sheldrick, 2008 ▶); molecular graphics: *PLATON* (Spek, 2009 ▶); software used to prepare material for publication: *SHELXL97*.

## Supplementary Material

Crystal structure: contains datablocks global, I. DOI: 10.1107/S1600536809049939/su2143sup1.cif

Structure factors: contains datablocks I. DOI: 10.1107/S1600536809049939/su2143Isup2.hkl

Additional supplementary materials:  crystallographic information; 3D view; checkCIF report

## Figures and Tables

**Table 1:** Hydrogen-bond geometry (Å, °)

*D*—H⋯*A*	*D*—H	H⋯*A*	*D*⋯*A*	*D*—H⋯*A*
N1′—H1′*N*⋯O1′	0.86 (2)	1.99 (2)	2.704 (2)	138 (2)
N1—H1*N*⋯O1	0.94 (2)	2.02 (2)	2.767 (2)	134 (2)
N2′—H2′*N*⋯O1^i^	0.90 (2)	1.96 (2)	2.850 (2)	167 (2)
N2—H2*N*⋯O1′	0.90 (2)	2.02 (2)	2.921 (2)	173 (2)
C9—H9⋯*Cg*1^ii^	0.95	2.79	3.496 (2)	132
C15′—H15′⋯*Cg*2	0.95	2.95	3.779 (2)	148
C19—H19⋯*Cg*5^iii^	0.95	2.79	3.595 (2)	143
C3′—H3′⋯*Cg*6^iv^	0.95	2.97	3.933 (2)	175

Symmetry codes: (i) 
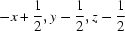
; (ii) 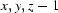; (iii) 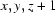; (iv) 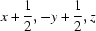. *Cg*1 is the centroid of ring I (C1–C6), *Cg*2 is the centroid of ring II (C7–C12), *Cg*5 is the centroid of ring II′ (C7′–C12′) and *Cg*6 is the centroid of ring III′ (C14′–C19′)

**Fig. 1.**
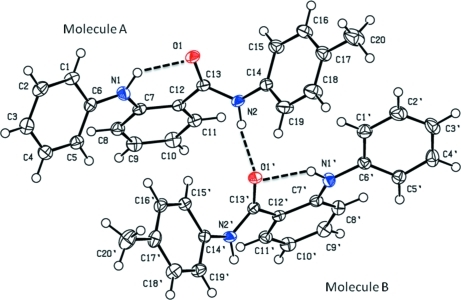
The molecular structure of the title compound, showing 30% probability displacement ellipsoids and the atomic numbering. Intra- and inter-molecular N—H···O hydrogen bonds are shown as dashed lines.

## supplementary crystallographic information

### Comment

In the past decade, the assessment of new hydrogen bonding patterns has received great attention due to their potential applications in biological, materials and supramolecular sciences, and crystal engineering. Here we report on the crystal structure of the title compound, a phenyl-amino-benzamide.

The molecular structure of the two independent molecules (A and B) of the title compound are illustrated in Fig. 1. The bond lengths and angles are within normal ranges. The conformation of the two independent molecules is slightly different. The dihedral angles between the central aromatic ring, II (C7—C12 molecule A, C7'-C12' molecule B), and rings I (C1—C6 molecule A, C1'-C6' molecule B) and III (C14—C19 molecule A, C14'-C19' molecule B), are 47.13 (9) and 89.36 (9)°, respectively, for molecule A, and 29.60 (9) and 70.72 (9)°, respectively, for molecule B. Rings I and III are inclined to one another by 86.57 (9)° in molecule A, and 64.59 (10)° in molecule B. The amide unit (N1/C7/O1 molecule A, N1'/C7'/O1' molecule B) lies out of the plane of rings II and III; the dihedral angles being 38.2 (2) and 52.2 (2)°, respectively, in molecule A, and 34.5 (2) and 36.4 (2)°, respectively, in molecule B. The molecular structure of each molecule is stabilized by a N—H···O intramolecular hydrogen bond, involving the amino H-atom and the benzamide carbonyl O-atom (Table 1).

In the crystal structure molecules are linked through N—H···O intermolecular hydrogen bonds, involving the benzamide carbonyl O-atom (O1 and O1') and the 4-methyl-Benzenamine amino H-atoms (H2'N and H2N) of the other molecule (Table 1), so forming chains propagating in direction [010]. In addition a number of C—H···π interactions are observed (Table 1). Footnote to Table 1: *Cg*1 centroid of ring I (= C1—C6); *Cg*2 centroid of ring II (= C7—C12); *Cg*5 centroid of ring II' (= C7'-C12'); *Cg*6 centroid of ring III' (= C14'-C19').

### Experimental

The title compound was prepared according to the reported procedures (Martín *et al.*, 2006; Charton *et al.*, 2006). Colourless single crystals suitable for X-ray diffraction were obtained by recrystallization from dichloromethane.

### Refinement

In the absence of significant anomalous dispersion effects, the Friedel pairs were merged. H atoms were placed in calculated positions, with N—H = 0.88–0.93 Å and C—H = 0.95 Å, and refined in riding mode, with *U*_iso_(H) = 1.2*U*_eq_(c,*N*).

### Crystal data

**Table:**

C_20_H_18_N_2_O	*D*_x_ = 1.247 Mg m^−^^3^
*M**_r_* = 302.36	Mo *K*α radiation, λ = 0.71073 Å
Orthorhombic, *P**n**a*2_1_	Cell parameters from 10095 reflections
*a* = 26.537 (3) Å	θ = 3.1–27.5°
*b* = 17.7337 (19) Å	µ = 0.08 mm^−^^1^
*c* = 6.8457 (7) Å	*T* = 93 K
*V* = 3221.6 (6) Å^3^	Block, colorless
*Z* = 8	0.50 × 0.40 × 0.33 mm
*F*(000) = 1280	

### Data collection

**Table:**

Rigaku SPIDER diffractometer	3913 reflections with *I* > 2σ(*I*)
Radiation source: Rotating Anode	*R*_int_ = 0.028
graphite	θ_max_ = 27.5°, θ_min_ = 3.1°
ω scans	*h* = −34→25
21180 measured reflections	*k* = −23→22
3989 independent reflections	*l* = −8→8

### Refinement

**Table:**

Refinement on *F*^2^	Primary atom site location: structure-invariant direct methods
Least-squares matrix: full	Secondary atom site location: difference Fourier map
*R*[*F*^2^ > 2σ(*F*^2^)] = 0.034	Hydrogen site location: inferred from neighbouring sites
*wR*(*F*^2^) = 0.074	H atoms treated by a mixture of independent and constrained refinement
*S* = 1.01	*w* = 1/[σ^2^(*F*_o_^2^) + (0.0338*P*)^2^ + 0.690*P*] where *P* = (*F*_o_^2^ + 2*F*_c_^2^)/3
3989 reflections	(Δ/σ)_max_ = 0.001
433 parameters	Δρ_max_ = 0.20 e Å^−^^3^
1 restraint	Δρ_min_ = −0.14 e Å^−^^3^

### Special details

**Table:**

Geometry. All e.s.d.'s (except the e.s.d. in the dihedral angle between two l.s. planes) are estimated using the full covariance matrix. The cell e.s.d.'s are taken into account individually in the estimation of e.s.d.'s in distances, angles and torsion angles; correlations between e.s.d.'s in cell parameters are only used when they are defined by crystal symmetry. An approximate (isotropic) treatment of cell e.s.d.'s is used for estimating e.s.d.'s involving l.s. planes.
Refinement. Refinement of *F*^2^ against ALL reflections. The weighted *R*-factor *wR* and goodness of fit *S* are based on *F*^2^, conventional *R*-factors *R* are based on *F*, with *F* set to zero for negative *F*^2^. The threshold expression of *F*^2^ > σ(*F*^2^) is used only for calculating *R*-factors(gt) *etc*. and is not relevant to the choice of reflections for refinement. *R*-factors based on *F*^2^ are statistically about twice as large as those based on *F*, and *R*- factors based on ALL data will be even larger.

### Fractional atomic coordinates and isotropic or equivalent isotropic displacement parameters (Å^2^)

**Table:**

	*x*	*y*	*z*	*U*_iso_*/*U*_eq_	
O1	0.25647 (5)	0.51964 (7)	0.6647 (2)	0.0269 (3)	
N1	0.15605 (6)	0.48341 (9)	0.6089 (3)	0.0241 (3)	
N2	0.30182 (5)	0.41608 (8)	0.5815 (2)	0.0215 (3)	
C1	0.09231 (7)	0.52823 (9)	0.8280 (3)	0.0243 (4)	
H1	0.1118	0.5725	0.8477	0.029*	
C2	0.04777 (7)	0.51875 (10)	0.9296 (3)	0.0287 (4)	
H2	0.0369	0.5567	1.0181	0.034*	
C3	0.01864 (7)	0.45450 (11)	0.9040 (3)	0.0294 (4)	
H3	−0.0121	0.4482	0.9736	0.035*	
C4	0.03524 (7)	0.39970 (10)	0.7750 (3)	0.0270 (4)	
H4	0.0157	0.3553	0.7570	0.032*	
C5	0.07986 (7)	0.40848 (10)	0.6713 (3)	0.0233 (4)	
H5	0.0906	0.3703	0.5832	0.028*	
C6	0.10904 (6)	0.47343 (9)	0.6968 (3)	0.0212 (4)	
C7	0.17326 (6)	0.45206 (9)	0.4353 (3)	0.0199 (4)	
C8	0.14034 (7)	0.43026 (9)	0.2865 (3)	0.0239 (4)	
H8	0.1051	0.4368	0.3036	0.029*	
C9	0.15820 (7)	0.39937 (10)	0.1154 (3)	0.0269 (4)	
H9	0.1350	0.3839	0.0175	0.032*	
C10	0.20968 (7)	0.39049 (11)	0.0835 (3)	0.0275 (4)	
H10	0.2218	0.3699	−0.0357	0.033*	
C11	0.24272 (7)	0.41228 (9)	0.2290 (3)	0.0232 (4)	
H11	0.2779	0.4068	0.2083	0.028*	
C12	0.22564 (6)	0.44210 (9)	0.4052 (3)	0.0202 (4)	
C13	0.26208 (6)	0.46334 (9)	0.5607 (3)	0.0202 (4)	
C14	0.34212 (6)	0.42888 (9)	0.7153 (3)	0.0213 (4)	
C15	0.36702 (7)	0.49770 (10)	0.7182 (3)	0.0274 (4)	
H15	0.3565	0.5372	0.6337	0.033*	
C16	0.40720 (7)	0.50894 (10)	0.8440 (3)	0.0290 (4)	
H16	0.4239	0.5563	0.8450	0.035*	
C17	0.42351 (7)	0.45242 (11)	0.9681 (3)	0.0295 (4)	
C18	0.39818 (8)	0.38430 (11)	0.9620 (4)	0.0389 (5)	
H18	0.4088	0.3447	1.0459	0.047*	
C19	0.35778 (8)	0.37202 (10)	0.8374 (3)	0.0320 (5)	
H19	0.3411	0.3246	0.8365	0.038*	
C20	0.46697 (9)	0.46438 (13)	1.1061 (4)	0.0473 (6)	
H20A	0.4553	0.4925	1.2208	0.057*	
H20B	0.4935	0.4930	1.0398	0.057*	
H20C	0.4803	0.4154	1.1475	0.057*	
O1'	0.30100 (5)	0.26650 (6)	0.4028 (2)	0.0237 (3)	
N1'	0.39641 (5)	0.23148 (8)	0.2976 (2)	0.0224 (3)	
N2'	0.25027 (5)	0.16713 (8)	0.3282 (2)	0.0216 (3)	
C1'	0.45616 (7)	0.30174 (10)	0.4848 (3)	0.0284 (4)	
H1'	0.4287	0.3287	0.5401	0.034*	
C2'	0.50487 (8)	0.31826 (11)	0.5432 (4)	0.0360 (5)	
H2'	0.5105	0.3558	0.6397	0.043*	
C3'	0.54540 (7)	0.28053 (11)	0.4622 (4)	0.0346 (5)	
H3'	0.5789	0.2932	0.4988	0.041*	
C4'	0.53645 (7)	0.22427 (11)	0.3276 (3)	0.0301 (4)	
H4'	0.5642	0.1979	0.2724	0.036*	
C5'	0.48774 (7)	0.20532 (10)	0.2708 (3)	0.0238 (4)	
H5'	0.4823	0.1650	0.1820	0.029*	
C6'	0.44695 (6)	0.24589 (9)	0.3451 (3)	0.0210 (4)	
C7'	0.37496 (6)	0.20358 (9)	0.1275 (3)	0.0196 (4)	
C8'	0.40260 (7)	0.18644 (10)	−0.0417 (3)	0.0234 (4)	
H8'	0.4380	0.1947	−0.0432	0.028*	
C9'	0.37921 (7)	0.15781 (10)	−0.2062 (3)	0.0262 (4)	
H9'	0.3988	0.1462	−0.3183	0.031*	
C10'	0.32753 (7)	0.14578 (10)	−0.2102 (3)	0.0255 (4)	
H10'	0.3117	0.1254	−0.3230	0.031*	
C11'	0.29945 (7)	0.16400 (9)	−0.0466 (3)	0.0219 (4)	
H11'	0.2640	0.1571	−0.0494	0.026*	
C12'	0.32198 (6)	0.19223 (9)	0.1227 (3)	0.0195 (3)	
C13'	0.29056 (6)	0.21259 (9)	0.2951 (3)	0.0200 (3)	
C14'	0.21651 (6)	0.17360 (9)	0.4875 (3)	0.0206 (4)	
C15'	0.20087 (6)	0.24292 (10)	0.5599 (3)	0.0239 (4)	
H15'	0.2127	0.2883	0.5024	0.029*	
C16'	0.16781 (7)	0.24530 (10)	0.7170 (3)	0.0258 (4)	
H16'	0.1579	0.2929	0.7678	0.031*	
C17'	0.14875 (7)	0.18014 (11)	0.8023 (3)	0.0272 (4)	
C18'	0.16414 (7)	0.11125 (10)	0.7252 (3)	0.0293 (4)	
H18'	0.1514	0.0658	0.7798	0.035*	
C19'	0.19773 (7)	0.10764 (10)	0.5704 (3)	0.0264 (4)	
H19'	0.2080	0.0600	0.5207	0.032*	
C20'	0.11217 (8)	0.18513 (13)	0.9705 (4)	0.0395 (5)	
H20D	0.0896	0.2282	0.9507	0.047*	
H20E	0.1309	0.1918	1.0927	0.047*	
H20F	0.0923	0.1386	0.9773	0.047*	
H1N	0.1801 (9)	0.5147 (13)	0.670 (4)	0.046 (7)*	
H2N	0.2992 (7)	0.3714 (12)	0.521 (4)	0.032 (6)*	
H1'N	0.3754 (8)	0.2536 (12)	0.375 (4)	0.033 (6)*	
H2'N	0.2491 (8)	0.1238 (12)	0.259 (4)	0.034 (6)*	

### Atomic displacement parameters (Å^2^)

**Table:**

	*U*^11^	*U*^22^	*U*^33^	*U*^12^	*U*^13^	*U*^23^
O1	0.0280 (7)	0.0196 (6)	0.0332 (8)	0.0032 (5)	−0.0046 (6)	−0.0098 (6)
N1	0.0214 (7)	0.0261 (7)	0.0248 (8)	−0.0018 (6)	0.0004 (7)	−0.0075 (7)
N2	0.0237 (7)	0.0163 (6)	0.0244 (8)	0.0026 (5)	−0.0035 (6)	−0.0065 (6)
C1	0.0310 (9)	0.0185 (8)	0.0235 (10)	0.0024 (7)	−0.0001 (8)	0.0004 (8)
C2	0.0353 (10)	0.0254 (9)	0.0253 (11)	0.0066 (7)	0.0054 (9)	0.0008 (8)
C3	0.0280 (10)	0.0323 (10)	0.0280 (11)	0.0030 (7)	0.0043 (8)	0.0078 (9)
C4	0.0271 (9)	0.0262 (9)	0.0276 (10)	−0.0016 (7)	−0.0042 (8)	0.0052 (8)
C5	0.0266 (9)	0.0220 (8)	0.0213 (9)	0.0031 (7)	−0.0062 (8)	0.0004 (7)
C6	0.0225 (8)	0.0217 (8)	0.0193 (9)	0.0042 (6)	−0.0022 (7)	0.0026 (7)
C7	0.0250 (9)	0.0163 (7)	0.0185 (9)	0.0015 (6)	−0.0016 (7)	0.0007 (7)
C8	0.0253 (9)	0.0222 (8)	0.0240 (10)	0.0015 (7)	−0.0037 (8)	0.0006 (8)
C9	0.0322 (10)	0.0284 (9)	0.0202 (10)	−0.0009 (7)	−0.0070 (8)	0.0011 (8)
C10	0.0348 (10)	0.0312 (9)	0.0166 (9)	−0.0002 (8)	0.0003 (8)	−0.0022 (8)
C11	0.0255 (9)	0.0207 (8)	0.0234 (10)	0.0003 (7)	0.0010 (7)	−0.0004 (8)
C12	0.0243 (8)	0.0155 (7)	0.0207 (9)	0.0008 (6)	−0.0014 (7)	−0.0001 (7)
C13	0.0234 (9)	0.0163 (7)	0.0209 (10)	−0.0006 (6)	0.0019 (7)	−0.0011 (7)
C14	0.0211 (8)	0.0216 (8)	0.0213 (9)	0.0005 (6)	−0.0002 (7)	−0.0055 (7)
C15	0.0290 (9)	0.0240 (8)	0.0293 (10)	−0.0037 (7)	−0.0030 (8)	0.0053 (8)
C16	0.0301 (10)	0.0253 (9)	0.0315 (11)	−0.0075 (7)	−0.0050 (9)	0.0015 (8)
C17	0.0285 (10)	0.0307 (9)	0.0294 (11)	−0.0047 (7)	−0.0072 (9)	0.0015 (9)
C18	0.0449 (12)	0.0287 (9)	0.0432 (13)	−0.0063 (8)	−0.0204 (11)	0.0112 (10)
C19	0.0377 (11)	0.0210 (9)	0.0374 (12)	−0.0057 (7)	−0.0112 (10)	0.0036 (9)
C20	0.0502 (14)	0.0425 (12)	0.0491 (15)	−0.0142 (10)	−0.0256 (12)	0.0115 (12)
O1'	0.0238 (6)	0.0183 (5)	0.0290 (7)	−0.0007 (5)	0.0005 (6)	−0.0077 (6)
N1'	0.0191 (7)	0.0266 (7)	0.0215 (8)	0.0003 (6)	0.0005 (7)	−0.0053 (7)
N2'	0.0233 (7)	0.0163 (7)	0.0251 (8)	−0.0018 (5)	0.0032 (7)	−0.0063 (6)
C1'	0.0297 (10)	0.0246 (9)	0.0308 (11)	0.0007 (7)	−0.0049 (9)	−0.0021 (9)
C2'	0.0394 (12)	0.0288 (10)	0.0397 (12)	−0.0048 (8)	−0.0155 (10)	−0.0016 (9)
C3'	0.0257 (10)	0.0350 (10)	0.0430 (13)	−0.0077 (8)	−0.0128 (10)	0.0125 (10)
C4'	0.0227 (9)	0.0360 (10)	0.0317 (11)	0.0035 (7)	0.0014 (8)	0.0141 (9)
C5'	0.0258 (9)	0.0229 (8)	0.0227 (9)	0.0014 (7)	0.0009 (8)	0.0043 (8)
C6'	0.0217 (8)	0.0202 (8)	0.0212 (9)	−0.0025 (6)	−0.0008 (7)	0.0042 (7)
C7'	0.0232 (8)	0.0167 (7)	0.0190 (9)	−0.0001 (6)	−0.0019 (7)	0.0011 (7)
C8'	0.0234 (9)	0.0259 (8)	0.0209 (9)	0.0008 (7)	0.0008 (7)	0.0015 (8)
C9'	0.0303 (9)	0.0305 (9)	0.0177 (9)	0.0030 (7)	0.0034 (8)	0.0002 (8)
C10'	0.0321 (10)	0.0252 (8)	0.0191 (9)	−0.0006 (7)	−0.0036 (8)	−0.0007 (8)
C11'	0.0235 (8)	0.0199 (8)	0.0223 (10)	−0.0005 (6)	−0.0026 (7)	0.0010 (7)
C12'	0.0230 (8)	0.0143 (7)	0.0213 (9)	0.0007 (6)	−0.0006 (7)	0.0003 (7)
C13'	0.0206 (8)	0.0166 (7)	0.0228 (9)	0.0022 (6)	−0.0027 (7)	−0.0006 (7)
C14'	0.0187 (8)	0.0220 (8)	0.0209 (9)	0.0007 (6)	−0.0015 (7)	−0.0049 (7)
C15'	0.0215 (9)	0.0211 (8)	0.0292 (11)	0.0007 (6)	0.0002 (8)	−0.0042 (8)
C16'	0.0248 (9)	0.0243 (8)	0.0283 (10)	0.0048 (7)	−0.0020 (8)	−0.0091 (8)
C17'	0.0269 (9)	0.0340 (9)	0.0208 (10)	0.0084 (7)	0.0008 (8)	−0.0011 (9)
C18'	0.0339 (10)	0.0279 (9)	0.0262 (10)	0.0027 (7)	0.0050 (9)	0.0022 (8)
C19'	0.0305 (10)	0.0219 (8)	0.0268 (11)	0.0018 (7)	0.0019 (8)	−0.0044 (8)
C20'	0.0448 (12)	0.0427 (12)	0.0309 (12)	0.0139 (9)	0.0115 (11)	0.0019 (10)

### Geometric parameters (Å, °)

**Table:**

O1—C13	1.235 (2)	O1'—C13'	1.239 (2)
N1—C7	1.389 (2)	N1'—C7'	1.388 (2)
N1—C6	1.396 (2)	N1'—C6'	1.404 (2)
N1—H1N	0.94 (2)	N1'—H1'N	0.86 (2)
N2—C13	1.354 (2)	N2'—C13'	1.358 (2)
N2—C14	1.426 (2)	N2'—C14'	1.416 (2)
N2—H2N	0.90 (2)	N2'—H2'N	0.90 (2)
C1—C2	1.381 (3)	C1'—C2'	1.385 (3)
C1—C6	1.396 (2)	C1'—C6'	1.398 (3)
C1—H1	0.9500	C1'—H1'	0.9500
C2—C3	1.388 (3)	C2'—C3'	1.383 (3)
C2—H2	0.9500	C2'—H2'	0.9500
C3—C4	1.385 (3)	C3'—C4'	1.379 (3)
C3—H3	0.9500	C3'—H3'	0.9500
C4—C5	1.389 (3)	C4'—C5'	1.391 (3)
C4—H4	0.9500	C4'—H4'	0.9500
C5—C6	1.399 (2)	C5'—C6'	1.396 (2)
C5—H5	0.9500	C5'—H5'	0.9500
C7—C8	1.396 (2)	C7'—C8'	1.404 (3)
C7—C12	1.416 (2)	C7'—C12'	1.420 (2)
C8—C9	1.378 (3)	C8'—C9'	1.382 (3)
C8—H8	0.9500	C8'—H8'	0.9500
C9—C10	1.392 (3)	C9'—C10'	1.388 (3)
C9—H9	0.9500	C9'—H9'	0.9500
C10—C11	1.382 (3)	C10'—C11'	1.383 (3)
C10—H10	0.9500	C10'—H10'	0.9500
C11—C12	1.393 (3)	C11'—C12'	1.397 (3)
C11—H11	0.9500	C11'—H11'	0.9500
C12—C13	1.487 (2)	C12'—C13'	1.490 (3)
C14—C19	1.374 (3)	C14'—C15'	1.389 (2)
C14—C15	1.388 (2)	C14'—C19'	1.392 (2)
C15—C16	1.385 (3)	C15'—C16'	1.389 (3)
C15—H15	0.9500	C15'—H15'	0.9500
C16—C17	1.384 (3)	C16'—C17'	1.390 (3)
C16—H16	0.9500	C16'—H16'	0.9500
C17—C18	1.383 (3)	C17'—C18'	1.392 (3)
C17—C20	1.506 (3)	C17'—C20'	1.508 (3)
C18—C19	1.387 (3)	C18'—C19'	1.386 (3)
C18—H18	0.9500	C18'—H18'	0.9500
C19—H19	0.9500	C19'—H19'	0.9500
C20—H20A	0.9800	C20'—H20D	0.9800
C20—H20B	0.9800	C20'—H20E	0.9800
C20—H20C	0.9800	C20'—H20F	0.9800
			
C7—N1—C6	127.70 (16)	C7'—N1'—C6'	130.64 (16)
C7—N1—H1N	113.2 (16)	C7'—N1'—H1'N	114.4 (16)
C6—N1—H1N	119.1 (16)	C6'—N1'—H1'N	113.0 (15)
C13—N2—C14	123.56 (15)	C13'—N2'—C14'	125.34 (16)
C13—N2—H2N	116.0 (13)	C13'—N2'—H2'N	116.4 (14)
C14—N2—H2N	119.7 (13)	C14'—N2'—H2'N	116.8 (14)
C2—C1—C6	120.75 (17)	C2'—C1'—C6'	120.72 (19)
C2—C1—H1	119.6	C2'—C1'—H1'	119.6
C6—C1—H1	119.6	C6'—C1'—H1'	119.6
C1—C2—C3	120.87 (18)	C3'—C2'—C1'	120.5 (2)
C1—C2—H2	119.6	C3'—C2'—H2'	119.7
C3—C2—H2	119.6	C1'—C2'—H2'	119.7
C4—C3—C2	118.63 (18)	C4'—C3'—C2'	118.95 (18)
C4—C3—H3	120.7	C4'—C3'—H3'	120.5
C2—C3—H3	120.7	C2'—C3'—H3'	120.5
C3—C4—C5	121.21 (17)	C3'—C4'—C5'	121.45 (19)
C3—C4—H4	119.4	C3'—C4'—H4'	119.3
C5—C4—H4	119.4	C5'—C4'—H4'	119.3
C4—C5—C6	120.04 (17)	C4'—C5'—C6'	119.63 (18)
C4—C5—H5	120.0	C4'—C5'—H5'	120.2
C6—C5—H5	120.0	C6'—C5'—H5'	120.2
C1—C6—N1	118.24 (16)	C5'—C6'—C1'	118.60 (17)
C1—C6—C5	118.50 (17)	C5'—C6'—N1'	124.26 (16)
N1—C6—C5	123.05 (16)	C1'—C6'—N1'	117.03 (16)
N1—C7—C8	121.94 (16)	N1'—C7'—C8'	123.72 (16)
N1—C7—C12	119.79 (16)	N1'—C7'—C12'	118.43 (16)
C8—C7—C12	118.27 (16)	C8'—C7'—C12'	117.85 (16)
C9—C8—C7	120.99 (17)	C9'—C8'—C7'	121.12 (16)
C9—C8—H8	119.5	C9'—C8'—H8'	119.4
C7—C8—H8	119.5	C7'—C8'—H8'	119.4
C8—C9—C10	121.07 (18)	C8'—C9'—C10'	121.06 (18)
C8—C9—H9	119.5	C8'—C9'—H9'	119.5
C10—C9—H9	119.5	C10'—C9'—H9'	119.5
C11—C10—C9	118.54 (18)	C11'—C10'—C9'	118.70 (18)
C11—C10—H10	120.7	C11'—C10'—H10'	120.7
C9—C10—H10	120.7	C9'—C10'—H10'	120.7
C10—C11—C12	121.60 (17)	C10'—C11'—C12'	121.66 (16)
C10—C11—H11	119.2	C10'—C11'—H11'	119.2
C12—C11—H11	119.2	C12'—C11'—H11'	119.2
C11—C12—C7	119.51 (16)	C11'—C12'—C7'	119.58 (17)
C11—C12—C13	120.31 (16)	C11'—C12'—C13'	120.30 (15)
C7—C12—C13	120.19 (16)	C7'—C12'—C13'	120.08 (16)
O1—C13—N2	122.23 (16)	O1'—C13'—N2'	122.34 (17)
O1—C13—C12	122.61 (15)	O1'—C13'—C12'	122.24 (15)
N2—C13—C12	115.15 (15)	N2'—C13'—C12'	115.41 (15)
C19—C14—C15	119.51 (17)	C15'—C14'—C19'	119.43 (17)
C19—C14—N2	120.05 (16)	C15'—C14'—N2'	122.37 (16)
C15—C14—N2	120.39 (17)	C19'—C14'—N2'	118.18 (15)
C16—C15—C14	120.12 (18)	C16'—C15'—C14'	119.47 (17)
C16—C15—H15	119.9	C16'—C15'—H15'	120.3
C14—C15—H15	119.9	C14'—C15'—H15'	120.3
C17—C16—C15	121.23 (17)	C15'—C16'—C17'	122.00 (17)
C17—C16—H16	119.4	C15'—C16'—H16'	119.0
C15—C16—H16	119.4	C17'—C16'—H16'	119.0
C18—C17—C16	117.53 (18)	C16'—C17'—C18'	117.62 (18)
C18—C17—C20	120.94 (19)	C16'—C17'—C20'	120.39 (17)
C16—C17—C20	121.53 (17)	C18'—C17'—C20'	121.99 (18)
C17—C18—C19	122.09 (19)	C19'—C18'—C17'	121.27 (18)
C17—C18—H18	119.0	C19'—C18'—H18'	119.4
C19—C18—H18	119.0	C17'—C18'—H18'	119.4
C14—C19—C18	119.51 (17)	C18'—C19'—C14'	120.18 (17)
C14—C19—H19	120.2	C18'—C19'—H19'	119.9
C18—C19—H19	120.2	C14'—C19'—H19'	119.9
C17—C20—H20A	109.5	C17'—C20'—H20D	109.5
C17—C20—H20B	109.5	C17'—C20'—H20E	109.5
H20A—C20—H20B	109.5	H20D—C20'—H20E	109.5
C17—C20—H20C	109.5	C17'—C20'—H20F	109.5
H20A—C20—H20C	109.5	H20D—C20'—H20F	109.5
H20B—C20—H20C	109.5	H20E—C20'—H20F	109.5
			
C6—C1—C2—C3	0.1 (3)	C6'—C1'—C2'—C3'	1.0 (3)
C1—C2—C3—C4	0.3 (3)	C1'—C2'—C3'—C4'	−2.4 (3)
C2—C3—C4—C5	−0.5 (3)	C2'—C3'—C4'—C5'	0.5 (3)
C3—C4—C5—C6	0.2 (3)	C3'—C4'—C5'—C6'	2.6 (3)
C2—C1—C6—N1	−175.43 (18)	C4'—C5'—C6'—C1'	−3.9 (3)
C2—C1—C6—C5	−0.4 (3)	C4'—C5'—C6'—N1'	−179.98 (17)
C7—N1—C6—C1	−157.58 (17)	C2'—C1'—C6'—C5'	2.2 (3)
C7—N1—C6—C5	27.7 (3)	C2'—C1'—C6'—N1'	178.51 (18)
C4—C5—C6—C1	0.2 (3)	C7'—N1'—C6'—C5'	−30.9 (3)
C4—C5—C6—N1	175.00 (18)	C7'—N1'—C6'—C1'	153.02 (18)
C6—N1—C7—C8	26.0 (3)	C6'—N1'—C7'—C8'	−2.7 (3)
C6—N1—C7—C12	−154.54 (17)	C6'—N1'—C7'—C12'	177.90 (16)
N1—C7—C8—C9	179.91 (16)	N1'—C7'—C8'—C9'	179.14 (16)
C12—C7—C8—C9	0.4 (3)	C12'—C7'—C8'—C9'	−1.5 (3)
C7—C8—C9—C10	−1.5 (3)	C7'—C8'—C9'—C10'	0.7 (3)
C8—C9—C10—C11	1.0 (3)	C8'—C9'—C10'—C11'	0.9 (3)
C9—C10—C11—C12	0.4 (3)	C9'—C10'—C11'—C12'	−1.6 (3)
C10—C11—C12—C7	−1.4 (3)	C10'—C11'—C12'—C7'	0.8 (3)
C10—C11—C12—C13	178.56 (16)	C10'—C11'—C12'—C13'	178.90 (15)
N1—C7—C12—C11	−178.50 (15)	N1'—C7'—C12'—C11'	−179.82 (15)
C8—C7—C12—C11	1.0 (2)	C8'—C7'—C12'—C11'	0.8 (2)
N1—C7—C12—C13	1.5 (2)	N1'—C7'—C12'—C13'	2.1 (2)
C8—C7—C12—C13	−179.01 (15)	C8'—C7'—C12'—C13'	−177.37 (15)
C14—N2—C13—O1	−3.9 (3)	C14'—N2'—C13'—O1'	−1.7 (3)
C14—N2—C13—C12	176.59 (16)	C14'—N2'—C13'—C12'	177.13 (15)
C11—C12—C13—O1	142.03 (18)	C11'—C12'—C13'—O1'	−144.84 (17)
C7—C12—C13—O1	−38.0 (2)	C7'—C12'—C13'—O1'	33.3 (2)
C11—C12—C13—N2	−38.5 (2)	C11'—C12'—C13'—N2'	36.4 (2)
C7—C12—C13—N2	141.49 (17)	C7'—C12'—C13'—N2'	−145.52 (16)
C13—N2—C14—C19	131.4 (2)	C13'—N2'—C14'—C15'	38.3 (3)
C13—N2—C14—C15	−51.1 (3)	C13'—N2'—C14'—C19'	−143.13 (18)
C19—C14—C15—C16	−0.3 (3)	C19'—C14'—C15'—C16'	1.7 (3)
N2—C14—C15—C16	−177.87 (17)	N2'—C14'—C15'—C16'	−179.80 (16)
C14—C15—C16—C17	0.3 (3)	C14'—C15'—C16'—C17'	−1.5 (3)
C15—C16—C17—C18	−0.1 (3)	C15'—C16'—C17'—C18'	0.3 (3)
C15—C16—C17—C20	−179.6 (2)	C15'—C16'—C17'—C20'	−178.89 (18)
C16—C17—C18—C19	−0.1 (4)	C16'—C17'—C18'—C19'	0.7 (3)
C20—C17—C18—C19	179.5 (2)	C20'—C17'—C18'—C19'	179.91 (19)
C15—C14—C19—C18	0.1 (3)	C17'—C18'—C19'—C14'	−0.5 (3)
N2—C14—C19—C18	177.73 (19)	C15'—C14'—C19'—C18'	−0.7 (3)
C17—C18—C19—C14	0.0 (4)	N2'—C14'—C19'—C18'	−179.27 (17)

### Hydrogen-bond geometry (Å, °)

**Table:**

*D*—H···*A*	*D*—H	H···*A*	*D*···*A*	*D*—H···*A*
N1'—H1'N···O1'	0.86 (2)	1.99 (2)	2.704 (2)	138 (2)
N1—H1N···O1	0.94 (2)	2.02 (2)	2.767 (2)	134 (2)
N2'—H2'N···O1^i^	0.90 (2)	1.96 (2)	2.850 (2)	167 (2)
N2—H2N···O1'	0.90 (2)	2.02 (2)	2.921 (2)	173 (2)
C9—H9···Cg1^ii^	0.95	2.79	3.496 (2)	132
C15'—H15'···Cg2	0.95	2.95	3.779 (2)	148
C19—H19···Cg5^iii^	0.95	2.79	3.595 (2)	143
C3'—H3'···Cg6^iv^	0.95	2.97	3.933 (2)	175

Symmetry codes: (i) −*x*+1/2, *y*−1/2, *z*−1/2; (ii) *x*, *y*, *z*−1; (iii) *x*, *y*, *z*+1; (iv) *x*+1/2, −*y*+1/2, *z*.
